# Toward the automatic detection of social interactions in gestating sows using image analysis data

**DOI:** 10.1093/jas/skaf249

**Published:** 2025-08-20

**Authors:** Anna Blanc, Alexandre Poissonnet, Johan Thomas, Valérie Courboulay, Mathieu Simon, Charlotte Gaillard

**Affiliations:** PEGASE, INRAE, Institut Agro, 35590, Saint-Gilles, France; IFIP Institut du Porc, 35740, Pacé, France; IFIP Institut du Porc, 35740, Pacé, France; IFIP Institut du Porc, 35740, Pacé, France; DILEPIX, 35000, Rennes, France; PEGASE, INRAE, Institut Agro, 35590, Saint-Gilles, France

**Keywords:** decision tree, distance, key points, movement speed, orientation

## Abstract

The identification of social interactions for group-housed gestating sows is crucial in order to monitor their welfare. They are key to understanding the social structure of the group and ensuring that disturbances, such as an abnormal frequency of aggression, are promptly detected. Assessing social interactions can be done through direct observation, manually recording the number and valence of interactions (positive or negative). This approach requires substantial human and financial resources. The study assesses the feasibility of using data produced by an image analysis software identifying sows’ postures and key points to automatically detect and classify social interactions. Two pens of 19 and 20 gestating sows each were video recorded from 00:00 to 02:00 on days 30 and 103 of two consecutive gestations. The videos were analyzed manually using the annotation system of the software in development. In total, 120 moments of positive interactions and 120 moments of negative interactions were annotated, including images before, during, and after the interaction. In addition, 120 moments without interactions (two sows not in contact) were annotated. The valence of the interaction (i.e., positive or negative), and for each interacting sow, the posture (i.e., standing, sitting, lying), and the coordinates of 3 key points (nose, neck, tail) were identified. Relative distances between sows, movement speeds, and individual distances traveled were calculated. Decision trees were performed to assess the relevance of these variables to detect an interaction. The start and end of an interaction were reliably detected with 88% accuracy using distances and postures. Interactions were characterized by a convergence of key points, spatial proximity, and at least one sow adopting a standing posture. The valence was effectively determined using the movement speeds and individual movements of sows during and after the interaction (respectively 74 and 80% accuracy). Negative interactions were characterized by faster movements and avoidance behavior. The performance in detecting sows’ orientation during interaction varied across categories (nose-nose, nose-neck, nose-tail, from 33% to 100%), and was particularly good for nose-tail interactions (94% F-score). This study shows the potential of the automatic image analysis software under development to detect and classify social interactions through post-processing analysis of key points and posture data.

## Teaser

This study demonstrates the potential of an automated image analysis software to detect and classify social interactions in group-housed pregnant sows via post-processing calculations. This technology could enable continuous welfare monitoring, reducing the need for manual observation while improving the detection of behavioral issues.

## Introduction

Since 2013, the European regulation has imposed to group gestating sows from the fourth week after insemination until the last week before farrowing (Council Directive, 2001). It allows greater freedom of movement and the expression of the species’ natural behaviors, such as exploration and positive social interactions, thus improving their welfare (Chapinal et al., 2010). However, in groups, pigs can also engage in negative social agonistic interactions. They are utilized by pigs when forming social hierarchy in the first days after grouping (Verdon and Rault, 2018). Agonistic social behaviors are also strongly linked to feeding strategies, as they are used to regulate access to a key resource (Boumans et al., 2018). Aggression can be influenced by a number of factors, such as the group size, feeding device, sow familiarity, or pen design (Arey and Edwards, 1998). An unusually large number of aggressions in a group may indicate a problem, such as with the ambient temperature (Abarnou et al., 2022), an unsuitable room structure, or feeding system malfunction to increased feed competition (Durand et al., 2023). These aggressions are a major welfare concern in group-housed sows, as they can lead to injuries and increased stress (Coutellier et al., 2007; Verdon et al., 2015).

Non-agonistic social interactions also play an important role in group dynamics (O’Malley et al., 2022). Their functions have not been clearly identified because they are rarely studied, although they may be involved in affiliation and recognition between individuals (Camerlink and Turner, 2013). For example, Martin et al. (2015) showed that piglets reared in an enriched environment showed greater socio-cognitive abilities, which resulted in improved welfare as shown by a reduction of stress and an increase of positive emotional states. The increasing proportion of socio-positive interactions, such as social nosing is likely to be beneficial to the individual and the group cohesion (Camerlink and Turner, 2013). Detecting socio-positive interactions would give a better understanding of the social dynamics of the group and a better group housing management, therefore improving sows’ welfare.

Assessing social interactions of pigs (e.g., animals involved, number, timing, and valence, that is, whether the interaction is positive or negative) is a challenge in environments where animals are in groups, such as gestating sows. Emotional valence in pig social behavior has been explored in a limited number of studies, highlighting both affiliative (positive) and agonistic (negative) interactions (Mendl et al., 2010). It requires observations, either direct or via video analysis. However, observation by an operator is time-consuming, cannot be done continuously, and is at risk of bias, which can affect the results (Tuyttens et al., 2014). Automatic image analysis using artificial intelligence seems to be an interesting and promising noninvasive alternative to detect social interactions. Digital image analysis uses machine learning to automatically detect specific information from images (Chen et al., 2021). The challenge of image analysis is to extract some of the available information automatically in order to reduce the manual workload, increase objectivity, and quantify changes that are too slight for the human eye (Oczak et al., 2014). Some studies have already focused on the automatic detection of aggressive interactions through digital image analysis in group-housed pigs (Oczak et al., 2014; Viazzi et al., 2014; Chen et al., 2017). In these studies, the detection of pixel movements is used to discriminate moments of high and medium aggression. In particular, Oczak et al. (2014) used an activity index, described as the comparison of the position of the animal in 2 successive images, and had good performances to discriminate a high and a medium aggression, as shown by an average accuracy of 99.2%. A similar method using pixel Motion History Image, for example, pixel intensity, was developed by Viazzi et al. (2014) with an average accuracy of 89%. The authors concluded that pig movement, characterized by animal speed and orientation relative to another individual (i.e., nose-nose contact, nose-tail, etc.) are important components of an interaction. This finding is supported by Chen et al. (2017), who used the acceleration data of the pigs involved in interactions and found that acceleration was higher during aggressive behaviors compared to other types of behaviors. They had an average accuracy of 96%. These studies have focused on intense agonistic behaviors such as aggression but do not consider lower-intensity agonistic behaviors such as threats or social-positive behaviors, which are valuable indicators of an animal’s state of welfare.

Gan et al. (2021) used the detection of key points on pre-weaning piglets’ noses and tails to characterize social interactions, relying on proximity and orientation data. They achieved favorable detection performances, distinguishing between social nosing, aggressive, and play behaviors with an overall precision of 92%. The identification of key points (nose, body, tail) during animal allowed for the detection of both aggressive and social-positive behaviors. This also provides robustness against deformation of the animals’ bodies depending on the camera angle, and against a difficult environment (Gan et al., 2021). To our knowledge, no such image analysis software has been developed to detect and characterize social interactions in a group of gestating sows.

For this project, an image analysis software is being developed to detect the activity of gestating sows continuously, in particular their posture (i.e., standing, lying, sitting) and their location by detecting the coordinates of 3 key points (i.e., nose, neck, tail). The aim of the present study is to evaluate the potential of an automated social interaction detection method based on these data produced by image analysis software. The variables of posture, distance, movement speed, and orientation between the sows will be calculated in order to detect interaction moments between two sows, determine their valence (positive or negative), and orientation (nose-nose, nose-neck, nose-tail).

## Material and Methods

### Animals and housing

The protocol for the experiment was reviewed and approved by the Ethics Committee in Animal Experimentation (Rennes, France, reference APAFiS #24663). The experiment was conducted in accordance with the French legislation for commercial pig production and experimental animal care. The experiment ran from July 2021 to December 2022 at the Pig Physiology and Phenotyping Experimental Facility (UE3P), Saint-Gilles, France (doi: 10.15454/1.5573932732039927E12).

**Figure 1.**
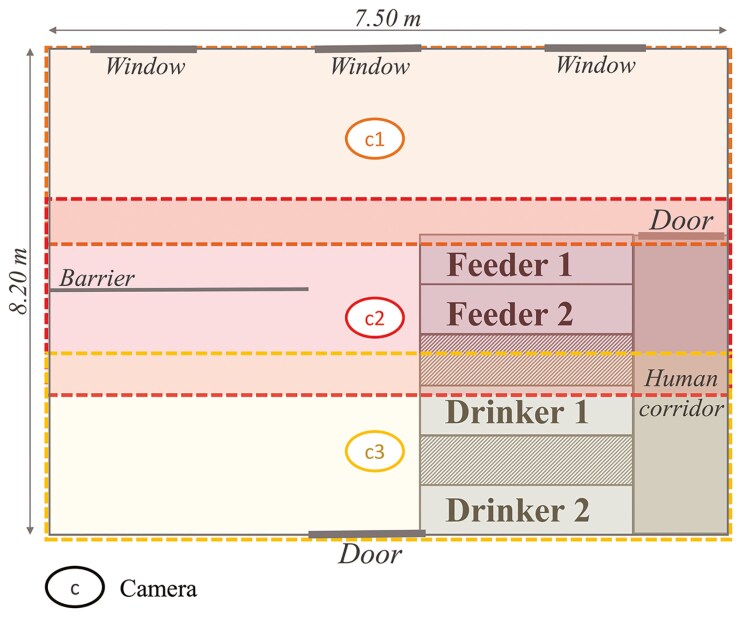
Gestating room with automatic feeders (Gestal, JYGA Technologies Inc., Quebec, Canada) and drinkers (ASSERVA, France), as well as the three cameras and their recording areas (with overlapping areas). Gestating room of 7.5m x 8m with a concrete floor on which straw was spread every day. The barrier consists of a metal half-wall with an openwork design to separate two resting areas.

Two batches of 19 and 20 Large White × Landrace sows were enrolled during two consecutive gestations, resulting in 4 groups. Three days after insemination, each group was housed in a gestation room of 7.5 m × 8 m with a concrete floor on which straw was spread every day. Each room was equipped with two automatic concentrate feeders (Gestal, JYGA Technologies, Inc., Quebec, Canada) and two automatic drinkers (ASSERVA, France) positioned side by side along a wall (Figure 1). These four automatons were capable of identifying each sow using an RFID tag.

Each sow received a daily feed ration based on her parity, body weight, and backfat thickness at insemination. The ration composition was adjusted daily, based on the lysine requirement of each individual, calculated with the InraPorc model adapted for the gestating sows (Dourmad et al., 2008; Gaillard et al., 2019). A daily and individual mixture of two diets (a diet with a high digestible lysine content of 8.5 g/kg, and a diet with a low digestible lysine content of 3.3 g/kg) was distributed by the feeders (13.14 MJ ME/kg). The start of a new feeding day was at midnight, and the feeders stayed open all day to allow all sows to enter the device freely.

Three dome cameras (Hikvision DARK FIGHTER, 1920 × 1080 resolution) were mounted strategically in each gestation room to ensure full coverage of the entire space (Figure 1) and provided continuous day and night recording. The cameras operated in a constant bandwidth mode, offering a video quality set to “medium,” with an image rate of 12 images per second (fps), which could reach up to 25 fps. The video encoding used the latest H.265 technology, ensuring efficient storage and high-quality footage. The cameras were positioned to optimize visibility and were able to record under both low-light and well-lit conditions, making them suitable for 24-h monitoring. Recordings were saved on external hard drives, and back ups were made weekly. To enable individual visual identification, each sow was marked weekly by a letter on her back using an animal marking crayon (RAIDEX).

### Observations and measurements

#### Manual observations

Videos analysis was carried out by a qualified observer trained in ethology and specifically in analyzing these types of videos and behaviors of interest. To assess intra-observer reliability, Cohen’s Kappa was calculated to determine the consistency of the observer’s behavior coding across different sessions. A Kappa value of 0.71 indicated substantial agreement, confirming the reliability of the observations. For each batch, 10 sows were chosen for observations. They were selected in order to represent the population as closely as possible (with different parities and weights). These individuals were observed on days 30 (early gestation, after hierarchy establishment) and 103 of gestation (late gestation, before transfer to the maternity pen), between 00:00 and 02:00 AM (the most active period in the room, as the new daily ration was made available at midnight). During this period, the social interactions (Table 1) of each observed sow were identified continuously, as well as the duration of the interactions (in seconds) and their valence (positive or negative). In total, across the 8 annotated periods (2 batches, 2 d per gestation, 2 gestation stages), 1,194 interactions were recorded (642 negative and 552 positive).

**Table 1.** Ethogram of the different behaviors considered.

Type of behavior	Description
***Postures***	
Standing	The sow stands on her 4 limbs. Only the hooves are in contact with the ground.
Lying	The sow has her body in full contact with the ground, either in lateral or sternal recumbency.
Sitting	The chest and flanks are off the ground, all or part of the hindlegs against the ground
***Social interactions***	Direct contact between an initiating sow’s nose a recipient sow. The initiating sow is standing, and the recipient can be in any posture.
*Positive interactions*	
Sniffing	The sow touches part of another sow’s body with her nose
Gently manipulating	A sow touches part of another sow’s body and moves her nose up and down on its body. This category includes licking (the sow’s tongue is out) and “gently pushes” (the tongue is not out, the sow gently pushes part of the other’s body with her nose).
*Negative interactions*	
Biting	The sow has open mouth contact with another sow’s head or body.
Kicking	The sow strikes another sow with her head or body, mouth closed.
Threatening	The sow makes a sudden movement of the head toward another sow, but no contact is established.
***No interaction***	
Lying side by side	Two sows are lying side by side with at least one part of their body in contact (excluding the nose)
Proximity	Two sows are in any posture (excluding both sows lying) and in relatively close proximity (visible via the same camera)

#### Software annotations

**Figure 2.**
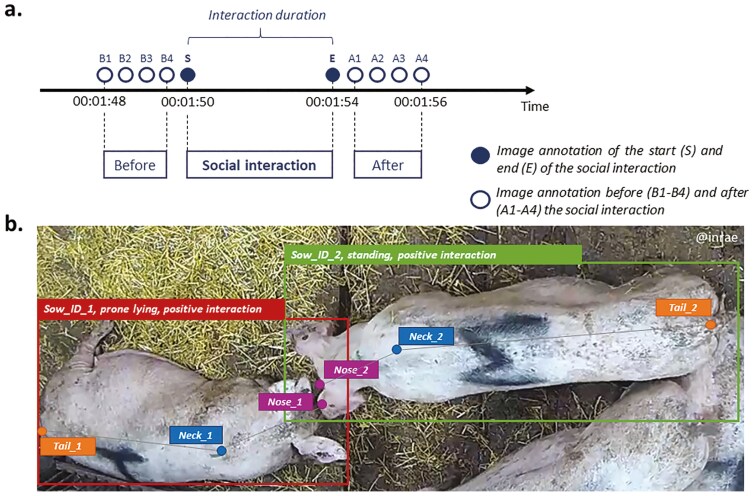
(a) Description of the image annotation method with an example of social interaction lasting 4 seconds, from 00:01:50 AM to 00:01:54 AM; (b) Picture of the annotation software with an example of positive nose-nose interaction between two sows.

Among the observed interactions, 120 positive and 120 negative interactions were randomly selected and specifically annotated using an annotation software (Dilepix Annotator 0.11.0., Rennes, France). The 120 interactions of each type were randomly selected, although care was taken to ensure that all 20 sows were equally represented in the new dataset to avoid the effect of one animal having a specific pattern. As we rely on contact between the sows’ key points to detect an interaction in our study, non-contact negative interactions (threats) have not been taken into account. Image by image, the two interacting sows were identified and outlined using a bounding box. The sows involved in the interaction were randomly designated as sow 1 and sow 2, without any particular distinction between the sow initiating the interaction and the sow receiving it. For each interaction, the image corresponding to the start of the interaction (S) and the image corresponding to the end of the interaction (E) were annotated, as well as 4 images before the interaction (B1-B4) and 4 images after the interaction (A1-A4) over an interval of 2 s (i.e., one image per half-second). In total, for each interaction, 10 images were annotated (Figure 2a.). These 10 images correspond to an interaction moment. For these 10 pictures, the sow’s posture (Table 1) was indicated, and the coordinates of three key-points (nose, neck, and tail, Figure 2b) were placed on the sow’s body by the operator. The nose key point was placed on the tip of the nose, the neck key point at the neck end between the two shoulders, and the tail key point at the insertion point between the tail and the rest of the body.

In addition to the 240 social interactions annotated, 120 moments of non-interactions were annotated to create a dataset with sows not interacting. To do so, in the same periods, 10 images of two sows not interacting were annotated every half-second in order to have as many images as during an interaction. Similarly to social interaction events, their posture and key points were annotated. The two non-interacting annotated sows were chosen to represent a range of possible configurations between individuals not engaged in social interaction, including being at a visible distance from one another (i.e., no body parts overlapping or in contact), lying side by side without nose interaction, or being in close proximity without displaying social behaviors (Table 1). Proximity was assessed visually based on body orientation and the absence of physical contact.

#### Calculations

From these image annotations created by the operator, a.csv file containing all image annotations was generated, with two lines per image, one for each annotated sow in the observed pair. The file included each sow’s posture, the coordinates (x and y) of the three key points (nose, neck, tail), whether the sows were interacting, and if so, the interaction valence (positive or negative). From this file, a new file was generated by combining the paired sows’ coordinates and postures into a single line and calculating variables for the three successive stages (i.e., before interaction, interaction, after interaction). Using R studio software version 2023.06.2 (R Core Team, 2023), variables were calculated:

For each sow, the posture (standing, sitting, lying) was recorded in each image. The proportion of time spent in each posture was calculated as:

Posture = (Number of images in posture)/(Total number of images)

These proportions were then combined to characterize the pair’s overall posture dynamics during the event (%standing, %sitting, %lying).

The distances between the different key points of the paired sows (sow 1 and sow 2) were calculated for each image using the Euclidean distance formula:

Distance (A, B) = √ (x_B − x_A)^2^ + (y_B − y_A)^2^

Where A and B represent key points (e.g., nose, neck, or tail), and x and y are the horizontal and vertical coordinates (in pixels) of the given point as extracted by the image analysis software. For instance, the distance between the two sows’ noses was computed as:

Distance (nose₁, nose₂) = √ [(nose_x₂ − nose_x₁)² + (nose_y₂ − nose_y₁)²]

This was repeated for multiple point combinations: nose1-nose2, nose1-neck2, nose2-neck1, nose1-tail2, and nose2-tail1.

For each pair, the average distance (AvgD) over the full sequence of images during an interaction (or non-interaction) was computed:

AvgD_point1_point2 = mean of Distance (point1, point2) over all images

The evolution of the distance between two key points over time was defined as the difference between their average distance in the first and the last image of the video sequence:

D_evolution = AvgD_last_image − AvgD_first_image

This metric provides an estimate of whether the two animals were moving closer together or further apart over the course of the observation. The duration of the interaction was computed in seconds, based on the number of images and the video image rate (12 fps). The speed of movement was calculated for each point-pair as:

Speed_point1_point2 = Distance traveled / Duration (in seconds)

Speed was first expressed in pixels per second, as the distance measurement was based on pixel coordinates, and then converted to meters per second (real-world calibration).

Lastly, the individual displacement of each sow was calculated by measuring the straight-line distance between the nose position in the first and last image of the sequence:

D_travelled_sow1 = √ [(nose_x_last − nose_x_first)² + (nose_y_last − nose_y_first)²]

D_travelled_sow2 = same calculation for sow 2

The individual distance traveled enables seeing any individual movements of flight, distance, or closeness to a particular individual. Therefore, for each interaction, 6 types of variables (average distance, distance evolution, individual distance traveled, speed, posture, and orientation) were recorded before, during, and after the interaction. For the non-interaction moments, the average distances, the distance evolution, the individual distances traveled, and the posture were calculated.

### Description of the Method

#### Three steps

The analyses were carried out using R studio software version 2023.06.2 (R Core Team, 2023). The first step consists of detecting the presence of an interaction between two sows, that is, when the sows start the contact, and when they end it. Therefore, to detect the start of the interaction, the four images before the interaction were considered, as well as the first image of the interaction (B1-B4 and S, Figure 2a), creating a dataset called “Before interaction.” To detect the end of the interaction, the four images after the interaction were considered, as well as the last image of the interaction (A1-A4 and E, Figure 2a), creating a dataset called “After interaction.” To differentiate between the presence and absence of interaction, this step considered 120 non-interactions moments and 120 interactions (positive and negative) chosen randomly to create a balanced dataset. The two categories are defined by the variable “Situation,” with the modalities “Interaction” and “No_interaction.” The analysis considered several variables described in Table 2.

**Table 2.** Description of the three steps method and the variables included to detect social interaction, its valence and orientation.

Step	Objective	Period considered	Predictor Variable	Explanatory variables
**Step 1**	Detecting the interaction start and end	Before and After	Situation (Interaction and no interaction)	Average distances between sows (AvgD_), Posture (%posture), Distances evolution (D_evolution), Individual distances traveled (D_travelled_)
**Step 2**	Detecting the interaction valence	Before, During and After	Interaction valence (Positive and negative)	Movement speeds between sows (Speed_), Posture (%posture), Individual distances traveled (D_travelled_)
**Step 3**	Detecting the interaction orientation	Before, During and After	Interaction orientation (nose-nose, nose-neck, nose-tail)	Average distances between sows (AvgD_), Distances evolution (D_evolution)

The second step of the method consists of detecting the valence of interaction (positive vs. negative Table 2) and identifying the period that enables optimum valence discrimination. Therefore, three periods were considered: before interaction (with images from B1 to B4 and S, Figure 2a) during interaction (e.g., the two images corresponding to the interaction, S and E), and after interaction (images from A1 to A4 and E). This step included the 240 interactions (120 positive and 120 negative), excluded the non-interactions, and considered the valence (positive or negative) as a variable to explain. The movement speed, postures, and distances traveled individually were considered to illustrate the movements of the sows (Table 2).

The third step consists of distinguishing the orientation of the interaction. The orientation of the interaction would provide more detailed information about the intentions behind the interactions (e.g., nose-nose positive interactions could indicate particular affinities between individuals) and possible consequences on welfare (e.g., negative nose-tail interactions could indicate tail biting, therefore leading to potential injury). Three periods were considered: before interaction (B1-B4 and S), during interaction (S and E), and after interaction (E and A1-A4).

This step also considered the 240 interactions (120 positive and 120 negative), excluded the non-interactions, and considered the interaction orientation as the variable to explain (Table 2).

#### Analysis method

For each step of the method, a principal component analysis (PCA) was performed on the explanatory variables to examine the relationships among them. The predictor variable was then projected onto the PCA to observe how it aligns with the principal components. This step shows the distribution of the data and enables a selection of variables for the next step by removing those that are highly correlated. In the context of PCA, a loading represents the contribution of each explanatory variable to the dimension.

Then, a classification was conducted using a regression tree (CART method from the rpart package, Therneau and Atkinson, 2022) model with the predictor variable as the response variable and the explanatory variables as predictors. In a decision tree, each internal node represents a combination of feature values, and each branch corresponds to a possible value or outcome. The hyperparameter chosen for the maximum depth of the tree was 3, as it gave the best performances (among 1 to 5). The model was trained on 80% of the dataset (training dataset) and then evaluated on the remaining 20% (test set) to assess its performance, with care taken to ensure that there were a sufficient number of observations for each modality in each data set.

The regression tree was tested on the test dataset (remaining 20%) to assess the reliability of the method. To evaluate the model’s reliability, a confusion matrix was generated by comparing the test results to the ground truth labels. The number of true positive, false negative, false positive, and true negative were respectively counted for each modality of the predictor variable. Specificity (1), sensitivity (2), precision (3), and F_score (4) were calculated for these modalities. Additionally, global accuracy (5) was computed to gauge the model’s performance.

(1) Specificity = Number of true negative/Number of false positive and true negative
(2) Sensitivity = Number of true positive/Number of true positive and false negative
(3) Precision = Number of true positive/Number of true positive and false positive
(4) F_score = 2*((Precision*Sensitivity)/(Precision + Sensitivity))
(5) Accuracy = Number of true positive and true negative/Total number of observations

## Results

### Step 1: Detecting the start and end of an interaction

#### Detecting the interaction start

##### PCA.

**Figure 3.**
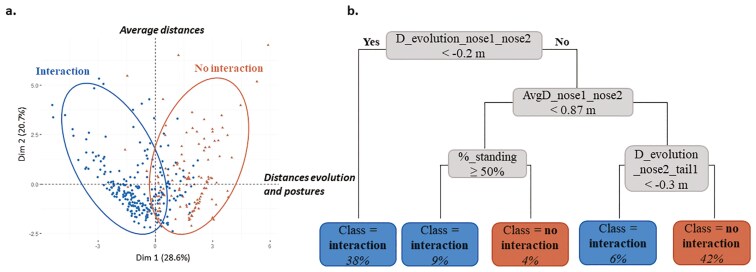
(a) Result of the Principal Component Analysis (PCA) performed on the 120 moments of interaction and 120 moments of no interaction “before interaction,” with dimensions 1 and 2; (b) Decision tree to predict the presence/absence of interaction on the training dataset “before interaction”. The percentage indicates the distribution of data in the branches of the tree

A PCA was performed on 120 moments of interaction and 120 moments of no interaction based on the 2 s preceding each event (period “before interaction”). The first two principal components explained 49.3% of the total variance (Figure 3a).

The first principal component accounted for 28.57% of the total variance. It was primarily influenced by the following variables:

- percentage of images with lying sows: loading = + 0.64,
- D_evolution_nose1_nose2: + 0.78,
- D_evolution_nose1_neck2: + 0.75,
- D_evolution_nose2_neck1: + 0.72
- percentage of images with standing sows: -0.63.

These results indicate that paired sows’ postures and their evolution in inter-individual distances contributed most to this component. We therefore labeled this dimension: “Distance evolution and postures.”

The second principal component accounted for 20.71% of the total variance. It was mainly influenced by:

- AvgD_nose1_nose2: + 0.78,
- AvgD_nose1_neck2: + 0.80,
- AvgD_nose2_neck1: + 0.72.

This component was there for labeled “Average distances”. Due to high collinearity among distance variables, D_evolution_nose1_neck2 and D_evolution_nose2_neck1 were excluded from the explanatory set used in the decision tree to avoid redundancy. Similarly, we removed AvgD_nose1_neck2 and AvgD_nose2_neck1.

##### Decision tree.

The decision tree (Figure 3b) classified situations as interactions or non-interactions using the remaining explanatory variables. A situation was classified as an interaction when D_evolution_nose1_nose2 was lower than −0.2 m, indicating a convergence between the noses of the paired sows during the 2 s preceding the interaction. When D_evolution_nose1_nose2 was higher than −0.2 m, a situation was still classified as interaction in the following cases:

- AvgD_nose1_nose2 was below 0.87 m and %standing above 50%, indicating that the sows were already in close proximity and at least one was active;
- AvgD_nose1_nose2 was above 0.87 m and D_travelled_nose2_tail1 below –0.3 m, suggesting movement toward the other sow despite greater initial distance.

These results suggest that the start of an interaction could either result from active convergence between sows (negative distance evolution) or from an established proximity combined with physical readiness to interact (standing posture). The overall classification accuracy was 88%, with all performance metrics (sensitivity, specificity, precision, *F*-score) exceeding 80% (Table 3).

**Table 3.** Performance metrics of modalities (sensitivity, specificity, precision, F1-score) and global performances (F-score, Accuracy) for each decision tree performed

		Number of observation	Metric for each modality				Global metric	
			Sensitivity	Specificity	Precision	F-score	F-score	Accuracy
**Step 1—Interaction**								
Before	Interaction	120	0.91	0.85	0.84	0.88	0.89	0.88
	No interaction	120	0.85	0.91	0.92	0.89		
After	Interaction	120	0.88	0.88	0.88	0.88	0.88	0.88
	No interaction	120	0.88	0.88	0.88	0.88		
**Step 2—Valence**								
Before	Positive	120	0.58	0.52	0.65	0.61	0.57	0.59
	Negative	120	0.52	0.57	0.52	0.52		
During	Positive	120	0.73	0.76	0.75	0.74	0.75	0.74
	Negative	120	0.76	0.73	0.74	0.75		
After	Positive	120	0.78	0.83	0.82	0.80	0.81	0.80
	Negative	120	0.83	0.78	0.79	0.81		
**Step 3—Orientation**								
Before	Nose-nose	119	0.81	0.60	0.63	0.71	0.70	0.67
	Nose-neck	68	0.38	0.83	0.55	0.44		
	Nose-tail	53	0.89	1.00	1.00	0.94		
During	Nose-nose	119	0.70	0.86	0.89	0.78	0.70	0.74
	Nose-neck	68	0.33	0.93	0.67	0.44		
	Nose-tail	53	0.86	0.76	0.92	0.89		
After	Nose-nose	119	0.77	0.79	0.88	0.82	0.74	0.74
	Nose-neck	68	0.60	0.82	0.50	0.55		
	Nose-tail	53	1.0	0.94	0.71	0.83		

#### Detecting the interaction end

##### PCA.

The first two principal components explained 50.7% of the total variance of the PCA performed after interaction. The first principal component accounted for 29.32% of the variance. It was positively influenced by:

- Percentage of images with standing sows: + 0.89
- D_evolution_nose1_nose2: + 0.58
- D_evolution_nose1_neck2: + 0.62
- D_evolution_nose2_neck1: + 0.60.

It also showed a strong negative loading for the percentage of images with lying sows: –0.90.

These results suggest that paired sows’ postural activity and divergence in inter-individual distances were key contributors to this component. This dimension was labeled: “Distance evolution and postures.”

The second principal component explained 21.44% of the variance. It was primarily influenced by AvgD_nose1_nose2 (+ 0.62). The dimension was labeled “Average distance nose-nose”. Because D_evolution_nose1_neck2 and D_evolution_nose2_neck1 were highly correlated with D_evolution_nose1_nose2, they were excluded from the explanatory variables in the decision tree to avoid redundancy.

##### Decision tree.

For the period after interaction, the decision tree classified a situation as an interaction based on specific patterns of divergence between paired sows. A situation was classified as an interaction in the following cases:

- D_evolution_nose1_nose2 was above 0.13 m and AvgD_nose1_nose2 below 2.5 m,
- D_evolution_nose1_nose2 was above 1.1 m.

These scenarios reflected a clear separation between the noses of the sows during the 2 s following the interaction. When D_evolution_nose1_nose2 was below 0.13 m, a situation was still classified as interaction in two cases:

- D_evolution_nose1_nose2 was higher than 0.14 m,
- D_evolution_nose2_nose1 was higher than 0.14 m.

These outcomes also indicated a post-interaction divergence between the paired sows. The classification model achieved an overall accuracy of 88%, with all performance metrics (sensitivity, specificity, precision, and F-score) also reaching 88% (Table 3).

### Step 2/Detecting the interaction valence

#### Before interaction

##### PCA.

A PCA was performed on moments preceding positive and negative interactions (*n* = 120 each). The first two principal components explained 74.4% of the total variance. The first principal component accounted for 50.7% of the total variance. It was positively influenced by:

- Speed_nose1_nose2: + 0.89
- Speed_nose1_neck2: + 0.83
- Speed_nose2_neck1: + 0.84
- Speed_nose1_tail2: + 0.54
- Speed_nose2_tail1: + 0.67

This principal component primarily represented the relative movement speeds between the paired sows and was labeled: “Speeds.” The second principal component accounted for 23.7% of the total variance. It contrasted the individual movement of the paired sows:

- D_travelled_sow1: –0.56
- D_travelled_sow2: + 0.71.

This component captured individual travel differences and was labeled: “Individual distances.” To reduce redundancy in the model, Speed_nose1_neck2 and Speed_nose2_neck1, which were highly correlated with Speed_nose1_nose2, were excluded from the explanatory variables used in the decision tree.

##### Decision tree.

The decision tree aimed to classify the valence of interaction (positive vs. negative) based on movement and posture indicators during the 2 s preceding interaction. An interaction was classified as negative in the following cases:

- Speed_nose1_nose2 was below 0.22 m/s, and the percentage of images showing both paired sows lying was below 50%
- Speed_nose1_nose2 was above 0.22 m/s and D_travelled_sow2 above 1.1 m.

These conditions suggest that reduced mutual movement or asymmetrical activity (e.g., one sow being highly mobile) tended to precede negative interactions. The decision tree model achieved a global accuracy of 59%, with performance metrics (sensitivity, specificity, precision, F-score) ranging from 0.52 to 0.65 (Table 3).

#### During interaction

##### PCA.

A PCA was performed on moments recorded during 120 positive and 120 negative interactions. The first two principal components explained a total of 71.9% of the variance. The first principal component accounted for 52.9% of the total variance and was positively influenced by:

- Speed_nose1_nose2: + 0.87

- Speed_nose1_neck2: + 0.79
- Speed_nose2_neck1: + 0.73
- Speed_nose1_tail2: + 0.74
- Speed_nose2_tail1: + 0.70
- D_travelled_sow1: + 0.62
- D_travelled_sow2: + 0.61

This principal component reflected the overall movement dynamics between and within sows, and was labeled: “Speeds and distances traveled individually.” The second principal component accounted for 19.1% of the total variance. It contrasted the sows’ individual displacements:

- D_travelled_sow1: + 0.52
- D_travelled_sow2: –0.50.

This second principal component was labeled “Distances traveled individually.” To avoid redundancy in the tree model, Speed_nose1_neck2 and Speed_nose2_neck1, which were highly correlated with Speed_nose1_nose2, were excluded from further analyses.

##### Decision tree.

The decision tree identified key variables distinguishing between positive and negative interactions during the interaction phase. An interaction was classified as negative in the following cases:

- D_travelled_sow1 was above 0.47 m.
- D_travelled_sow1 was below 0.47 m and Speed_nose1_tail2 was above 0.069 m/s or Speed_nose1_nose2 was above 0.3 m/s.

These conditions suggest that greater speed and individual displacement, particularly from one sow, were indicative of negative interactions, possibly reflecting avoidance or chasing behavior. The model achieved a global accuracy of 74%, with all performance metrics (sensitivity, specificity, precision, *F*-score) exceeding 70% (Table 3).

#### After interaction

##### PCA.

**Figure 4.**
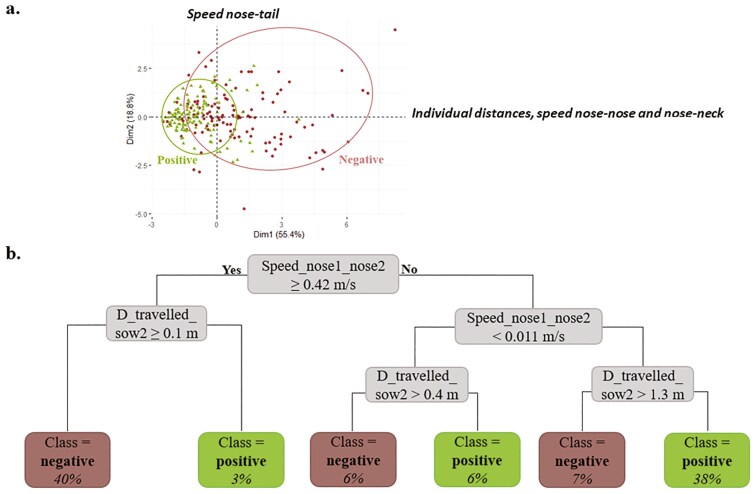
(a) Result of the Principal Component Analysis (PCA) performed on the 120 positive and 120 negative interactions “after interaction,” with dimensions 1 and 2; (b) Decision tree to predict the valence of interaction on the training dataset “after interaction.” The percentage indicates the distribution of data in the branches of the tree

The first two principal components explained 74.2% of the total variance from the PCA performed on moments of positive and negative interactions after interaction (120 moments each, Figure 4a.). The first principal component accounted for 55.4% of the total variance. It was positively influenced by:

- Speed_nose1_nose2: + 0.93
- Speed_nose1_neck2: + 0.91
- Speed_nose2_neck1: + 0.87
- D_travelled_sow1: + 0.62
- D_travelled_sow2: + 0.76.

This component captured both the movement between paired sows (nose-nose and nose-neck speeds) and individual displacements. It was labeled: “Individual distances and speeds (nose-nose and nose-neck).” The second principal component accounted for 18.8% of the total variance. It reflected opposite contributions from:

- Speed_nose2_tail1: + 0.69
- Speed_nose1_tail2: –0.59.

The second principal component was thus labeled “Speed nose-tail.” To avoid redundancy in the decision tree, Speed_nose1_neck2 and Speed_nose2_neck1, which were highly correlated with Speed_nose1_nose2, were excluded from the explanatory variables.

##### Decision tree.

The decision tree (Figure 4b) classified negative interaction after interaction using a combination of Speed_nose1_nose2 and D_travelled_sow2. A situation was classified as negative in the following cases:

- Speed_nose1_nose2 was above 0.42 m/s and D_travelled_sow2 above 0.1 m,
- Speed_ nose1_nose2 was below 0.011 m/s and D_travelled_sow2 above 0.4 m,
- Speed_ nose1_nose2 was between 0.011 and 0.42 m/s, and D_travelled_sow2 above 1.3 m.

These thresholds suggest that higher inter-individual speed, or substantial displacement of one sow, often occurred in negative interactions, potentially indicating escape or pursuit behaviors. The model reached a global accuracy of 80%, with all performance metrics (sensitivity, specificity, precision, *F*-score) ranging from 78% to 83% (Table 3).

### Step 3: Detecting the interaction orientation

#### Before interaction

##### PCA.

A PCA was conducted on moments of positive and negative interactions before interaction (120 moments each). The first two principal components explained 53.54% of the total variance. The first principal component accounted for 32.53% of the total variance. It was positively influenced by:

- D_evolution_nose1_nose2: + 0.76
- D_evolution_nose1_neck2: + 0.75
- D_evolution_nose2_neck1: + 0.74.

This component captured how distances between body parts evolved over time and was thus labeled: “Distances evolution.” The second principal component accounted for 21.01% of the total variance. It reflected contrasting contributions:

- AvgD_nose1_neck2: + 0.73
- AvgD_nose1_nose2: + 0.67
- AvgD_nose2_tail1: –0.77.

It was thus labeled “Average distances.”

##### Decision tree.

The decision tree classified interactions before they occurred into three orientation types (nose-nose, nose-neck, nose-tail), based on combinations of average distances and distance evolution. An interaction was classified as:

- nose-nose oriented when AvgD_nose1_nose2 was below 1.9 m and D_evolution_nose1_nose2 below -0.034 m.
- nose-neck oriented when AvgD_nose1_nose2 was below 1.9 m, D_evolution_nose1_nose2 above -0.034 m and AvgD_nose2_tail1 above 1.8 m, or when AvgD_nose1_nose2 was between 1.9 m and 2.2 m.
- nose-tail oriented when AvgD_nose1_nose2 was below 1.9 m, D_evolution_nose1_nose2 above -0.034 m, and AvgD_nose2_tail1 below 1.8 m, or when AvgD_nose1_nose2 was above 2.2 m.

This tree structure indicates that spatial proximity and approach dynamics between specific body parts prior to interaction are predictive of the type of orientation during the interaction.

The model achieved a global accuracy of 67%, with performance metrics ranging between 38% and 100% (Table 3).

#### During interaction

##### PCA.

The first two principal components explained 47.55% of the total variance from the PCA performed on the 120 moments of positive interactions and 120 moments of negative interactions during interaction. The first principal component accounted for 22.53% of the total variance. It was positively influenced by:

- AvgD_nose1_nose2: + 0.72,
- AvgD_nose1_neck2: + 0.77

It was negatively influenced by AvgD_nose2_tail1 (−0.58). This first principal component was thus labeled as “Average distances nose1-nose2, nose1-neck2, nose2-tail1.” The second principal component accounted for 18.10% of the total variance. It reflected contrasting contributions:

- AvgD_nose1_nose2: + 0.55,
- AvgD_nose2_neck1: + 0.80,
- AvgD_nose1_tail2: −0.69.

This second principal component was labeled “Average distances nose1-nose2, nose2-neck1, nose1-tail2.”

##### Decision tree.

An interaction was classified:

- nose-nose oriented when AvgD_nose1_nose2 was below 0.75 m,
- nose-tail oriented when AvgD_nose1_nose was below 1.7 m,
- nose-neck oriented when AvgD_nose1_nose2 was between 0.75 and 1.7 m.

The global accuracy was 74%, and the performance metrics varied between 33 and 93% (Table 3).

#### After interaction

##### PCA.

**Figure 5.**
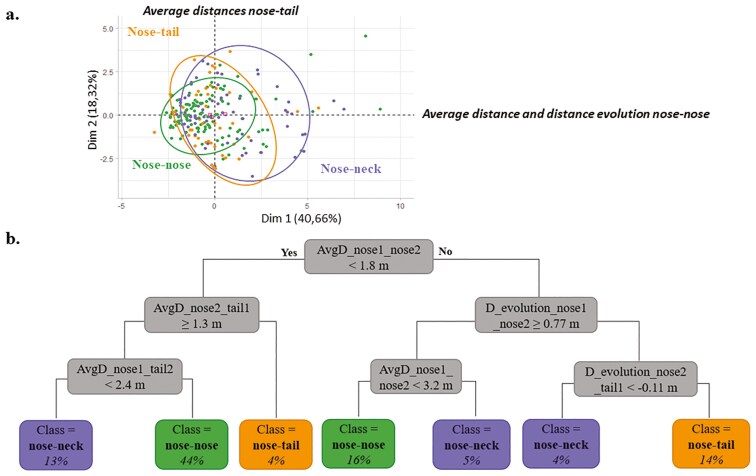
(a) Result of the Principal Component Analysis (PCA) performed on the 119 nose-nose, 68 nose-neck, and 53 nose-tail oriented interactions “after interaction,” with dimensions 1 and 2; (b) Decision tree to predict the orientation of interaction on the training dataset “after interaction.” The percentage indicates the distribution of data in the branches of the tree

The first two principal components explained 58.98% of the total variance from the PCA performed on the 120 moments of positive interactions and 120 moments of negative interactions after interaction (Figure 5a). The first principal component accounted for 40.66% of the total variance. It was positively influenced by:

- AvgD_nose1_nose2: + 0.83,
- AvgD_nose1_neck2: + 0.75,
- AvgD_nose2_neck1: + 0.72,
- D_evolution_nose1_nose2: + 0.82,
- D_evolution_nose1_neck2: + 0.81,
- D_evolution_nose2_neck1: + 0.81.

The first principal component was labeled: “Average distance and distance evolution nose-nose.” The second principal component accounted for 18.32% of the total variance and reflected contrasting contributions:

- AvgD_nose1_tail2: -0.77,
- AvgD_nose2_tail1: + 0.80.

The second principal component was labeled: “Average distances nose-tail.” To avoid redundancy with AvgD_nose1_nose2, AvgD_nose1_neck2, and AvgD_nose2_neck1 were removed from the explanatory variables used for the decision tree construction. Similarly, D_evolution_nose1_neck2 and D_evolution_nose2_neck1 were removed for the same reason.

##### Decision tree.

The decision tree (Figure 5b) showed that an interaction was classified nose-nose oriented when:

- AvgD_nose1_nose2 was below 1.8 m, AvgD_nose1_tail2 above 2.4 m and AvgD_nose2_tail1 above 1.3 m.
- AvgD_nose1_nose2 was between 1.8 m and 3.2 m, and D_evolution_nose1_nose2 above 0.77 m, reflecting an increasing distance between the two individuals.

It was classified nose-neck oriented when:

- AvgD_nose1_nose2 was below 1.8 m, AvgD_nose1_tail2 below 2.4 m and AvgD_nose2_tail1 above 1.3 m.
- AvgD_nose1_nose2 was above 3.2 m, and D_evolution_nose1_nose2 above 0.77 m.
- AvgD_nose1_nose2 was above 1.8 m, D_evolution_nose1_nose2 below 0.77 m, and D_evolution_nose2_tail1 below 0.11 m.

It was classified nose-tail oriented when:

- AvgD_nose1_nose2 was below 1.8 m and AvgD_nose2_tail1 below 1.3 m.
- The AvgD_nose1_nose2 was below 1.8 m, D_evolution_nose1_nose2 below 0.77 m, and D_evolution_nose2_tail1 above 0.11 m.

The global accuracy was 74%, and the performance metrics varied between 50% and 100% (Table 3).

## Discussion

The aim of this study was to evaluate a method to automatically detect social interactions between gestating sows using image analysis data containing data on the sows’ posture and key points coordinates. Using postures, distances between sows’ key point and the evolution of these distances over time showed good performances in detecting the start and end of a social interaction between two sows. The detection of the interaction valence also showed good results using movement speeds and individual movements, particularly after the interaction. Performances in detecting the orientation were heterogeneous depending on orientation, but showed promising results regardless of the period tested, particularly for nose-tail orientation detection.

## Variables Used as Indicators of Social Interactions

Using variables such as distance traveled between sows, average distance between sows, and posture recorded two seconds before the interaction turned out to be an effective approach for detecting the start of a social interaction (global accuracy of 88%). Sows interact when there is a movement toward each other, whether it is nose-nose or nose-tail oriented. The posture of the sows is also important to detect an interaction. However, it will be important to distinguish between lying on the side and prone lying in future studies. Indeed, lying on the side is a state of total relaxation and inactivity, while prone lying is a posture in which sows can interact with each other (Nicolaisen et al., 2019). It would be interesting to investigate whether social interactions between a standing sow and a lying sow occur when the lying sow is on her chest, suggesting a symmetric interaction involving both sows, or when the lying sow is on her side, which might indicate an approach initiated by the standing sow and an asymmetric interaction. The end of an interaction was also detected with satisfying accuracy and resulted in the sows moving away from each other. These information sheds light on the dynamics of sow movements during a social interaction, allowing us to distinguish between two sows actively interacting and two sows in close proximity without interacting.

The second step was to differentiate a positive interaction from a negative one, and to evaluate which period in the interaction was the most effective in distinguishing the two categories, before during and after the interaction. The movement speeds between the two sows and the individual distances traveled allowed the interaction valence discrimination with a similar accuracy when using images during or after the interaction (74% and 80% respectively), while the use of images before interaction was less effective. Negative interactions were characterized by a higher movement speed between the two sows. This result aligns with previous studies using automatic image analysis to detect aggressive behaviors of fattening pigs and showing greater acceleration and intensity of movement during aggression (Oczak et al., 2014; Viazzi et al., 2014; Chen et al., 2017). It is however interesting to see that the individual distances traveled also played an important role in the discrimination of social interactions, as they were greater during negative ones. This can be translated either as a pursuit by the sow initiating the interaction or as avoidance by the sow receiving it, which are typical behaviors of the species during an aggression (Clouard et al., 2024). It is supported by the work of Gan et al. (2021) who used the individual movement of piglets’ nose between images to discriminate social nosing from aggressive/playing behavior (global precision around 92%). This could also explain why the period after interaction showed the highest performance, as more significant sow movements occur at the end of the interaction than before sows get in contact.

The decision trees created still rely on the fictive identification given to the sow (1 or 2); it means that we do not have the information of which sow initiates the social behavior and which receives it. Having the identity of the initiating sow and the receiving sow could be interesting in order to have information on the nature of the behavior that would explain this high individual movement (avoidance or pursuit). Particularly in a context of agonistic interactions, we could characterize a particular behavioral pattern for a sow aggressing a conspecific and another pattern for a sow receiving aggression, and eventually determine the social profile of sows on an individual scale.

The performances on the detection of interaction orientation were heterogeneous according to the modalities considered but were similar regardless of the time period. Among the three orientations analyzed, the nose-neck orientation had the lowest performance. In fact, nose-neck interactions have a broader interaction surface considered compared to nose-nose and nose-tail, as they include all contacts on sows’ body (except for the head and the tail). Additionally, given that the neck key point is closer to the nose than the tail, there may be a confusion between nose-nose and nose-neck categories. It would be interesting to expand the dataset used to train decision trees to see if detection performance can be improved. The nose-tail category was the most accurately detected, with most performance metrics above 80% over the three periods considered, and despite a lower number of observations than other orientations. In gestating pens, nose-tail interactions are most commonly vulva bites that may induce injuries (Van Putten and Van De Burgwal, 1990). It is interesting to note that these interactions can be reliably detected with our method, as they could be used to monitor sow health by specifically identifying individuals that have experienced such interactions. This will require cross-referencing the valence of the interaction with its orientation. Specific orientations may be indicative of specific intentions behind the behavior. For example, authors have shown that social nosing (positive nose-nose contacts) can be used for individual recognition and would allow the establishment of affiliative links between individuals (Camerlink and Turner, 2013; Portele et al., 2019). Having information on both the orientation and valence would provide a more nuanced understanding of group interactions and allow for the detection of potential disruptions in the group’s social dynamics.

## The Method Devised

One goal of this study was to investigate the feasibility of using key points and sow postures to detect social interactions using post-processing based on sow movement and distance variables. To the best of our knowledge, this study is the first to aim at detecting both positive and negative social interactions in group-housed gestating sows using this method. For example, some studies focused on the detection of individual activity in lactating sows (lying, standing, eating, drinking; Lao et al., 2016; Yang et al., 2020), or on the detection at group-level of activity in gestating sows (Durand et al., 2023) but none has addressed the individual detection of activities in a group of pregnant sows. Other studies used automatic image analysis to detect aggressive behavior in piglets (Oczak et al., 2014; Viazzi et al., 2014; Chen et al., 2017), or to automatically detect tail biting (Liu et al., 2020). Oczak et al. (2014) and Viazzi et al. (2014) used bounding boxes around the piglets to determine the intensity of the animals’ movement, which gave information about the aggression intensity. Chen et al. (2017) considered the two piglets in agonistic interaction as a unique unit, and the acceleration of this unit enabled them to classify aggression according to its intensity. This method gave them a high accuracy in classifying the intensity of aggression (96% on average for Chen et al., 2017), but it is not suitable for detecting more subtle behaviors such as nosing. Indeed, this approach fails to provide information about the relative orientation of the two interacting animals (Wutke et al., 2021). However, sow’s nose is the main body part involved in the social behaviors (Gan et al., 2021). The use of key points is relatively recent, with some studies using it to detect social interactions in piglets (Gan et al., 2021; Wutke et al., 2021). Positioning key points on the animal provides more detailed information about the points of contact between the animals (head-head or head-tail; Wutke et al., 2021). Gan et al. (2021) used it to differentiate social nosing from playing and aggressive behaviors by detecting the head and the tail of fattening pigs, and had a global precision of 92%. They used a similar method as the present study to detect interactions, as their method was split in two steps: distinguishing interaction from non-interaction first, and then distinguishing play/aggressive behavior from social nosing. With head and tail key points as well on fattening pigs, a global precision of 95% for detecting head-head and head-tail contacts was achieved as well as the establishment of pen social network (Wutke et al., 2021). The method tested in the present study is new evidence that image analysis, in particular the use of key points, can detect specific social behavior. Then, by using the software currently under development for posture detection and vectorization of each sow in the pen over a given time period, we will be able to establish the number of social interactions, thereby providing automated monitoring of the group’s social behavior.

The present study tested a post-processing detection method using activity and key point data from image analysis. Although the performances are lower than other methods (overall accuracy of 75% vs. 96% for Chen et al., 2017; 89% for Viazzi et al., 2014), the results are promising and demonstrate that social interactions between gestating sows can be detected with high accuracy, without training a specific neural network on social interactions. With improved performance, this method will enable continuous monitoring of sow interactions; linked to other variables such as location in the room, eating behavior, and time spent in each posture, we will be able to monitor sows’ daily behavior continuously and automatically, and thus detect any health problems or malfunctions in the gestation room.

## Conclusion

The start and end of an interaction can be reliably detected based on the sows’ proximity, distance, and posture variables. Adding movement speed and individual distances will help capture the rapid movement of the sows during and after a negative interaction, allowing the discrimination between positive and negative interactions. By increasing the number of interactions annotated in each category and annotating the images during the interaction, we can develop a reliable and robust method for determining an interaction between two sows in a group, by using key points extracted from image analysis data.

## Abbreviations

AvgD_nose1_nose2: Average distance between the noses of the sows
AvgD_nose1_neck2: Average distance between the nose of sow 1 and the neck of sow 2
AvgD_nose2_neck1: Average distance between the nose of sow 2 and the neck of sow 1
AvgD_nose1_tail2: Average distance between the nose of sow 1 and the tail of sow 2
AvgD_nose2_tail1: Average distance between the nose of sow 2 and the tail of sow 1
D_evolution_nose1_nose2: Evolution of the distance over time between the noses of the sows
D_evolution_nose1_neck2: Evolution of the distance over time between the nose of sow 1 and the neck of sow 2
D_evolution_nose2_neck1: Evolution of the distance over time between the nose of sow 2 and the neck of sow 1
D_evolution_nose1_tail2: Evolution of the distance over time between the nose of sow 1 and the tail of sow 2
D_evolution_nose2_tail1: Evolution of the distance over time between the nose of sow 2 and the tail of sow 1
Speed_nose1_nose2: Movement speed between the noses of the sows
Speed_nose1_neck2: Movement speed between the nose of sow 1 and the neck of sow 2
Speed_nose2_neck1: Movement speed between the nose of sow 2 and the neck of sow 1
Speed_nose1_tail2: Movement speed between the nose of sow 1 and the tail of sow 2
Speed_nose2_tail1: Movement speed between the nose of sow 2 and the tail of sow 1
D_travelled_sow1: Distance traveled over time by sow 1
D_travelled_sow2: Distance traveled over time by sow 2
%standing: Percentage of images that sows spend in standing posture
%lying: Percentage of images that sows spend in lying posture
